# Automated evaluation of consistency within the PubChem Compound database

**DOI:** 10.1038/sdata.2019.23

**Published:** 2019-02-19

**Authors:** Hesam Dashti, Jonathan R. Wedell, William M. Westler, John L. Markley, Hamid R. Eghbalnia

**Affiliations:** 1 Department of Medicine, Brigham and Women’s Hospital and Harvard Medical School, Boston, Massachusetts 02215, USA; 2 National Magnetic Resonance Facility at Madison and BioMagResBank, Department of Biochemistry, University of Wisconsin Madison, Madison, Wisconsin 53706, USA

**Keywords:** Computational biology and bioinformatics, Data integration, Cheminformatics

## Abstract

Identification of discrepant data in aggregated databases is a key step in data curation and remediation. We have applied the ALATIS approach, which is based on the international chemical shift identifier (InChI) model, to the full PubChem Compound database to generate unique and reproducible compound and atom identifiers for all entries for which three-dimensional structures were available. This exercise also served to identify entries with discrepancies between structures and chemical formulas or InChI strings. The use of unique compound identifiers and atom nomenclature should support more rigorous links between small-molecule databases including those containing atom-specific information of the type available from crystallography and spectroscopy. The comprehensive results from this analysis are publicly available through our webserver [http://alatis.nmrfam.wisc.edu/].

## Introduction

Organic compounds with low molecular weights (usually less than 1,000 Da) are commonly categorized as small molecules. Small molecules are major targets for drug and biomarker discoveries^1,2^. For example, more than 60% of approved drugs in the 2017 United States Food and Drug Administration list are small molecules. The availability of accurate data about small molecules, including chemical, physical, and biomedical properties, is indispensable to many fields of endeavor. As the host to more than 94 million small molecule entries, PubChem^3^ is the most important resource for retrieving these types of metadata. The vast amount of information archived in PubChem, generated either internally or aggregated from about 2,000 external resources and databases [ftp://ftp.ncbi.nlm.nih.gov/pubchem/RDF/source/], serves as the central hub for inquiries related to small molecules. The accuracy of the archived and cross-referenced data in PubChem is a critical factor in the reproducibility of biomedical research studies that utilize this database.

Other databases contain essential data on the physical and chemical properties (e.g., mass spectrometry and nuclear magnetic resonance spectroscopy databases) and biological and toxicological properties (e.g., the Chemical Entities of Biological Interest (ChEBI)^4^) of small molecules. PubChem offers pre-aggregated data from several of these archives. However, the process of identifying and collating information on a specific compound from different data sources can be a challenging problem, partly owing to the absence of a system for unique compound identification that can provide an “anchor” for linking entries across different reference databases^5^. This impediment becomes evident when attempts are made to create cross-references linking the physical and chemical properties of the compounds archived in the ever-expanding number of databases containing experimental data on small molecules (e.g., BMRB^6^, HMDB^7^, PDB ligand expo [http://ligand-expo.rcsb.org/], METLIN^8^, MassBank of North America [http://mona.fiehnlab.ucdavis.edu/]) with reference databases such as PubChem.

In order to forge valid links in aggregated data and to detect and remediate discrepancies, a vital first step is to implement unique and reproducible identifiers for compounds and their constituent atoms. We recently created a software technology called ALATIS (atom label assignment tool using InChI string)^5^ that takes the structure file of a small molecule as input and produces the international chemical shift identifier (InChI)^9^ as the unique compound name, and further expands this identifier to uniquely label all constituent atoms of the compound. The ALATIS software program utilizes the “inchi-1” program (https://www.inchi-trust.org/, InChI version 1, software version 1.04) to generate standard InChI strings for input compounds. ALATIS utilizes the Open Babel^10^ software package to interconvert structure file formats and also to project 2D structures to 3D structures when needed. We recently used ALATIS in analyzing several databases: BMRB, HMDB, PDB Ligand Expo, and a small subset (0.05%) of PubChem entries. The results revealed the presence of non-standard InChI strings, inconsistent atom labeling, and inaccurate cross-references among the databases^5^.

In this study, we have expanded the ALATIS analysis to the entire PubChem database. The substantial computing resources needed for this work were provided through the enterprise-level computing platform of NMRbox^11^. The ALATIS results for the entire PubChem content have provided insights about the current state of this important resource. Most notably, the results provide unique and reproducible compound and atom identifiers associated with PubChem entries for use in improved curation and more accurate data retrieval.

## Results

**Figure 1:**
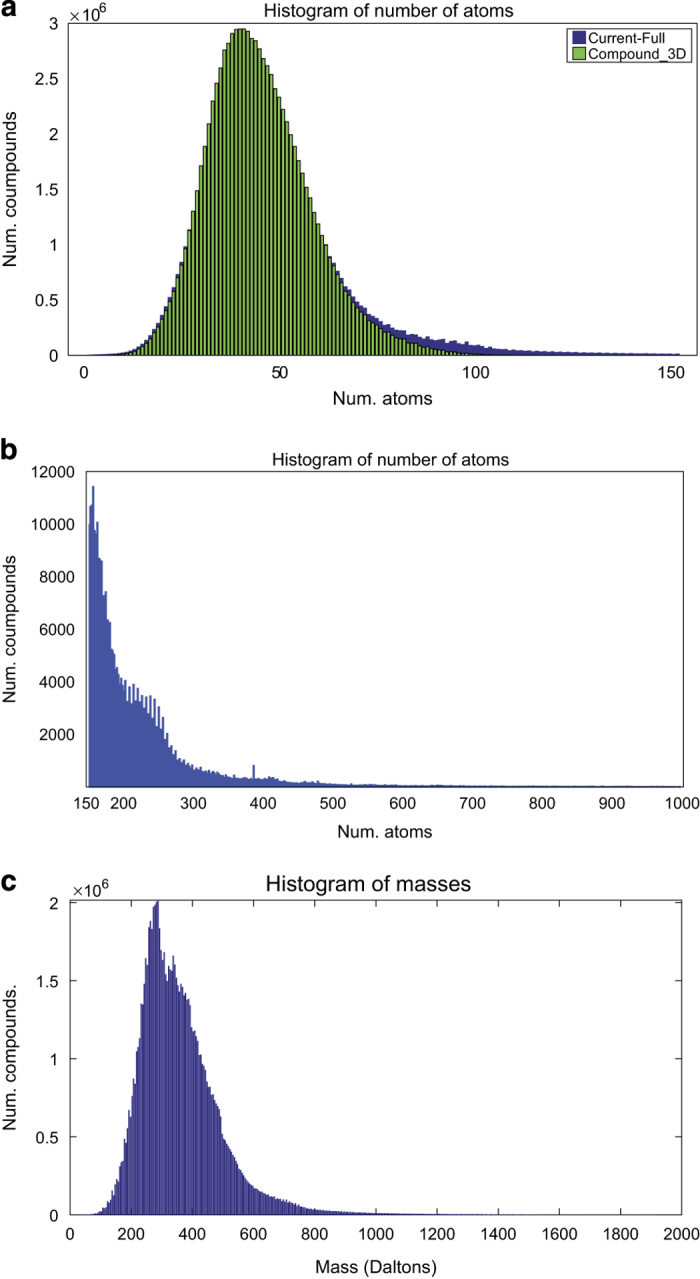
Histograms displaying the number of atoms and masses of entries in PubChem. The x-axis of histograms (**a**) and (**b**) represents the number of atoms in a compound, and the y-axis indicates the number of compounds with the corresponding number of atoms. (**a**) Histogram of masses for compounds with fewer than 152 atoms: those for “Current-Full” entries (2D structures) are shown in blue, and those for the “Compound_3D” entries are shown in green. The 152-atom cutoff was chosen based on the maximum number of atoms in compounds in the “Compound_3D” dataset. (**b**) Counts for compounds with >152 atoms. PubChem contains no 3D structure information for these compounds. (**c**) Histogram of masses of compounds as reported in the SDF files of PubChem “Current-Full” entries. Most of the compounds in the database had masses less than 1,000 Da; however, 11, 550 compounds had mass higher than 2,000 Da (not shown in (**c**)) – for example, PubChem CID 23393956 reported the exact mass of 59,745.256 Da.

We downloaded two sets of archived PubChem structure files on the twentieth of December 2017: (i) the “Current-Full” dataset consisting of 94,201,188 entries with their corresponding two-dimensional (2D) structures stored in SDF^12^ format, and (ii) the “Compound_3D” dataset consisting of 91,699,620 entries with their corresponding three-dimensional (3D) structures stored in SDF format. The “Current-Full” dataset was needed because it contains metadata that are not available in the “Compound_3D” files. More than 2.5 million entries in the PubChem did not have a 3D structure file. Interestingly, all compounds with more than 152 atoms did not have 3D structures (Fig. 1).

In order to probe the correctness of atom chirality, we processed the Compound_3D dataset with ALATIS software. This step generated unique identifiers for more than 91 million compounds and their constituent atoms (Data Citation 1). The output for each entry consisted of: (i) structure files in SDF, PDB, and XYZ formats containing ALATIS-based identifiers (labels) for all atoms, (ii) a map linking the input atom labels to the unique atom labels, (iii) a file containing a standard InChI string as the unique compound identifier (called ‘inchi.inchi’), (iv) two text files, named ‘warnings.txt’ and ‘error.txt’, that contain warnings or errors related to the ALATIS analysis of a particular compound, and (v) a comma-separated values (CSV) file, named ‘meta_data.csv’, containing the metadata associated with that entry. The metadata file contains, in addition to the PubChem compound identifier (CID), molecular formula, weight, and exact mass as reported by PubChem, the corresponding standard InChI string as generated by ALATIS. To facilitate side-by-side comparison of results, including comparison of input 3D structures and ALATIS output structures annotated with unique atom identifiers, we have generated a web page for each compound, which includes download links to all the data. We used the software Jmol [Jmol: an open-source Java viewer for chemical structures in 3D. http://www.jmol.org/] to create displays of the 3D structures. The unique compound and atom identifiers, along with information associated with PubChem entries, can be accessed through the ALATIS website [http://alatis.nmrfam.wisc.edu]. Users can query the search engine on this website with a PubChem CID or a compound name to retrieve the corresponding ALATIS output.

We used the ALATIS-curated data to analyze the consistency of the data stored for each entry in PubChem. Note that the synonyms and metadata are archived separately from the 3D structure files: synonyms are located at [ftp://ftp.ncbi.nlm.nih.gov/pubchem/Compound/Extras/CID-Synonym-filtered.gz] and that the metadata are stored as part of SDF files archived in “Current-Full” dataset [ftp://ftp.ncbi.nlm.nih.gov/pubchem/Compound/CURRENT-Full/SDF/]. The synonyms were used in creating a user-friendly search engine on the ALATIS webserver. The metadata were needed for the subsequent consistency analysis. We highlight below the two major outcomes of our study.

### Inconsistency between the archived 3D structures and formulas

**Figure 2:**
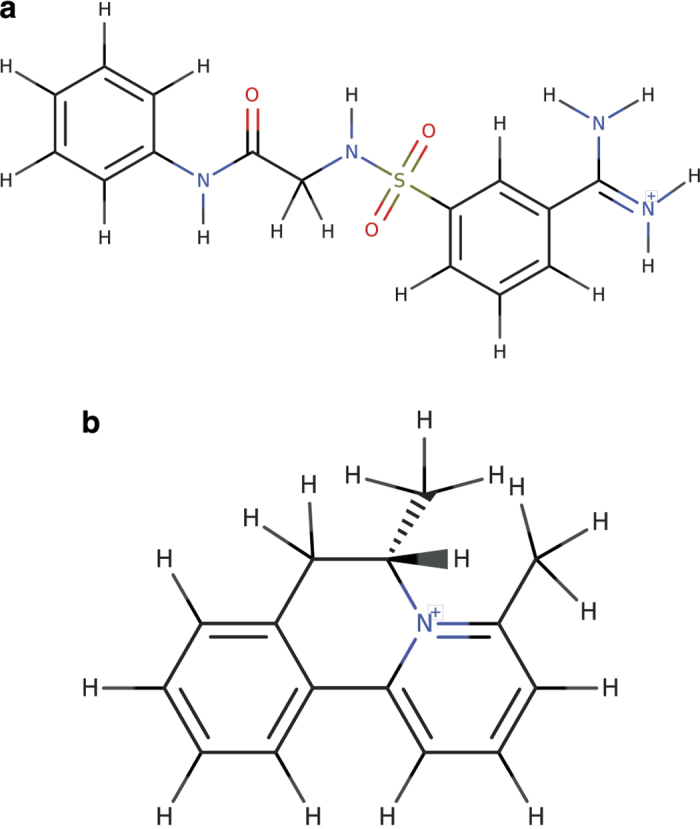
Examples of PubChem entries with charged chemical formulas. (**a**) PubChem CID 91929631. The archived chemical formula for this entry in PubChem is C_15_H_17_N_4_O_3_S^+^. This formula indicates 17 hydrogen atoms in the positively charged compound. However, the core parent structure of this compound contains only 16 hydrogen atoms; the additional hydrogen results from protonation of the compound in its charged form. The ALATIS formula for this compound “C15H16N4O3S” shows the correct atom composition with 16 hydrogen atoms, and the additional hydrogen is indicated in the corresponding InChI string (layer “/p”) “InChI = 1 S/C15H16N4O3S/c16-15(17)11-5-4-8-13(9-11)23(21,22)18-10-14(20)19-12-6-2-1-3-7-12/h1-9,18 H,10H2,(H3,16,17)(H,19,20)/**p + 1**” [http://alatis.nmrfam.wisc.edu/pubchem/91929631]. (**b**) PubChem CID 91124997. The chemical formula for this compound in PubChem is (C_15_H_16_N^+^). In this case, the positive charge arises from the quaternary nitrogen, and the correct composition of the compound contains 16 hydrogen atoms, which matches the formula in PubChem. The ALATIS formula for this compound shows the 16 hydrogen atoms (C15H16N), and the charge is represented by layer (“/q”) in the corresponding standard InChI string “InChI = 1 S/C15H16N/c1-11-6-5-9-15-14-8-4-3-7-13(14)10-12(2)16(11)15/h3-9,12 H,10H2,1-2H3/**q + 1**/t12-/m0/s1” [http://alatis.nmrfam.wisc.edu/pubchem/91124997].

The chemical formula of a compound archived in PubChem normally follows the Hill convention^13^ and represents the core parent structure of the compound^9^. However, the PubChem archive includes 1,239,752 charged chemical formulas, where charges are denoted by a symbol at the end of the chemical formula. The core parent structure of a compound indicates the composition of the compound before imposing any charges, through the addition or subtraction of hydrogen atoms. As illustrated by the examples in Fig. 2, it is not always possible to determine the core parent structure of a compound from its charged chemical formula. This is because, rather than resulting from the addition or subtraction of protons, the charge could be intrinsic to the covalent structure of the compound. Thus, large-scale computational processing and curation of the database could lead to inconsistent or ambiguous results in identifying the atom compositions of the compounds. This problem can be addressed by utilizing standard InChI strings. The formula layer of standard InChI strings provides the composition of the core parent of a compound, and the net charge (“/q”) and protonation (“/p”) layers of InChI strings represent compounds charges. This separation of charges from formulas facilitates extraction of the precise number of atoms in a compound’s structure file or chemical formula, as well as indicating the types of charges associated with the compound. We have produced a complete list of PubChem CIDs with charged chemical formulas, along with their corresponding ALATIS formulas in Hill format as extracted from standard InChI strings. These data are available on the ALATIS website.

### Inconsistency between the archived 3D structures and InChI strings

We compared the deposited PubChem InChI strings to those generated by ALATIS (ALATIS utilizes InChI program v. 1.04 [http://www.inchi-trust.org/download/104/InChI_TechMan.pdf]). Standard InChI strings represent unique compound identifiers that can be used for cross-referencing entries from different databases^5^. These strings consist of several layers of information, including compound formulas, covalent connectivity between heavy atoms, the number of hydrogen atoms associated with heavy atoms, a layer to represent chirality, and other layers associated with isotopically labeled atoms and compound charges^9^. We used ALATIS to process the 3D structure files deposited in PubChem, and flagged entries for which the corresponding deposited InChI strings failed to match those reported by ALATIS. Table 1 shows different categories of these flagged PubChem entries. In this table, the ‘Atom connectivity’ category reports the number of entries flagged because of discrepancies in (a) covalent connectivity between heavy atoms (reported in “/c” layer of InChI strings) or (b) the number of assigned hydrogen atoms to the heavy atoms (“/h” layer of InChI strings). The ‘Charge’ category reports the number of flagged entries that represent different (de)protonation (“/p” layer of InChI) or intrinsic covalent charges (“/q” layer). The ‘Stereochemistry’ category shows the number of entries that have been flagged because of discrepancies in their (a) “/b” layer of InChI strings that reports sp^2^ double bond stereochemistry of the compounds, or (b) InChI “/t” layer that reports orientations of chiral centers. We note that a compound could be flagged and reported in multiple categories. Overall, our analyses flagged 32,036,565 entries (about 33% of the PubChem entries with 3D structures) as having a discrepancy between its archived InChI string and that generated from the corresponding 3D structure by ALATIS. Improper representation of stereochemistry was the most common reason for discrepancy, followed by charge, and atom connectivity (Table 1). Complete lists of these flagged entries are reported on our website [http://alatis.nmrfam.wisc.edu/databases].

We provide below examples from the three categories of flagged inconsistencies.

#### (a) Inconsistency in atom connectivity

**Figure 3:**
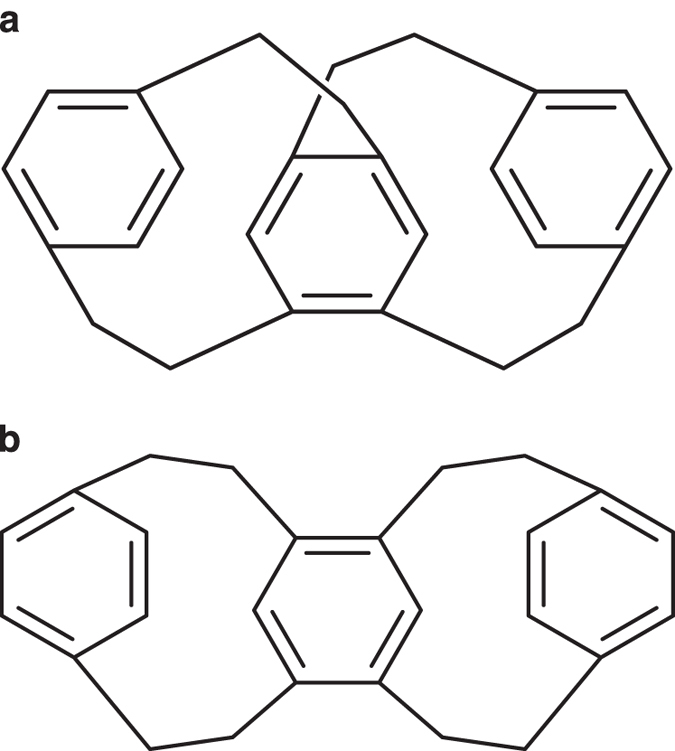
Inconsistency in the heavy atom connectivity layer. ALATIS generated standard InChI strings for 3D structures in PubChem, and by comparing these InChI strings with the deposited InChI strings in the database, we identified compounds whose connectivity layers did not match. This figure shows an example of such a discrepancy for PubChem CID 12300268. (**a**) Representation of the deposited 3D structure for the entry. (**b**) Representation from the deposited InChI string. We note that the covalent bonds are different between the two structures. The standard InChI string of the archived 3D structure: InChI = 1 S/C26H26/**c1-2-20-4-3-19(1)9-13-23-17-26-16-12-22-7-5-21(6-8-22)11-15-25(23)18-24(26)14-10-20**/h1-8,17-18 H,9-16H2, and the deposited InChI string: InChI = 1 S/C26H26/**c1-2-20-4-3-19(1)9-13-23-17-24(14-10-20)26-16-12-22-7-5-21(6-8-22)11-15-25(23)18-26**/h1-8,17-18 H,9-16H2. The bold-font InChI layers indicate the discrepancies between the connectivity of heavy atoms in the two InChI strings.

As noted above, the layers “/c” and “/h” in the standard InChI string represent the connectivity of heavy atoms and the number of associated hydrogen atoms to the heavy atoms, respectively. The PubChem entry shown in Fig. 3 illustrates a case in which the 3D structure file and the deposited InChI strings represent distinct covalent bonds between heavy atoms. Correct identification of 3D structure is essential in functional investigations of compounds, and this category of inconsistency could lead to erroneous conclusions.

#### (b) Inconsistency in charge distribution

**Figure 4:**
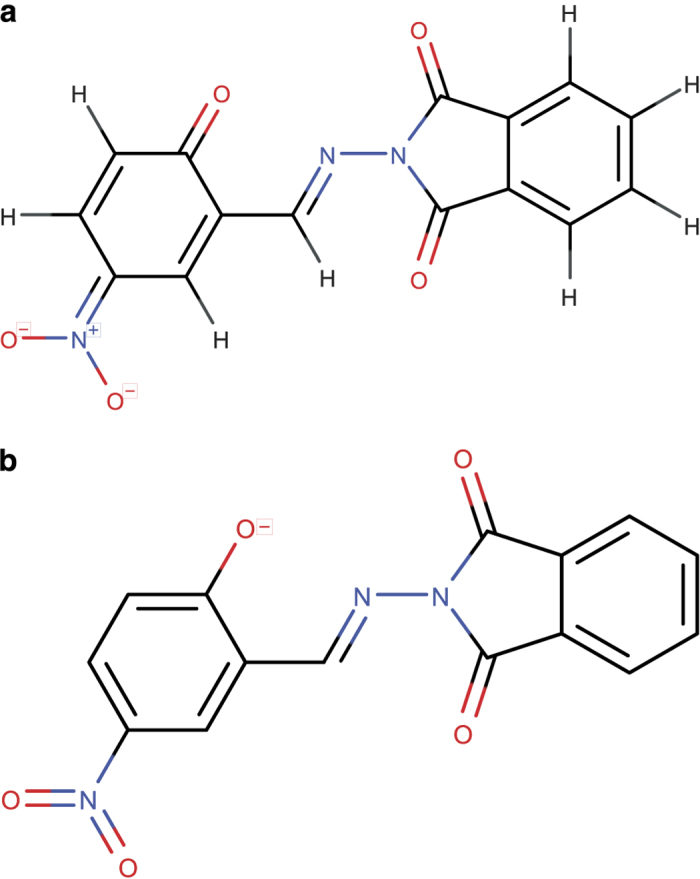
Inconsistency in charge. (**a**) Representation of the 3D structure file archived for PubChem CID 2179800. (**b**) Structural representation of the InChI string stored for that compound: “InChI = 1 S/C15H9N3O5/c19-13-6-5-10(18(22)23)7-9(13)8-16-17-14(20)11-3-1-2-4-12(11)15(17)21/h1-8,19 H/**p-1**”. The standard InChI string for the structure in (**a**) structure file reported by ALATIS is “InChI = 1 S/C15H8N3O5/c19-13-6-5-10(18(22)23)7-9(13)8-16-17-14(20)11-3-1-2-4-12(11)15(17)21/h1-8H/**q-1**”.

As mentioned above, distinct charges due to (de)protonation or intrinsic covalent charges of compounds are represented in the “/p” and “/q” layers of InChI strings. The flagged PubChem entries in this category are ones in which the archived 3D structure and InChI strings represent different charge states. Figure 4 shows an example from this category.

#### (c) Inconsistency in stereochemistry

##### (c.1) Inconsistency in double bond sp^2^ stereochemistry

**Figure 5:**
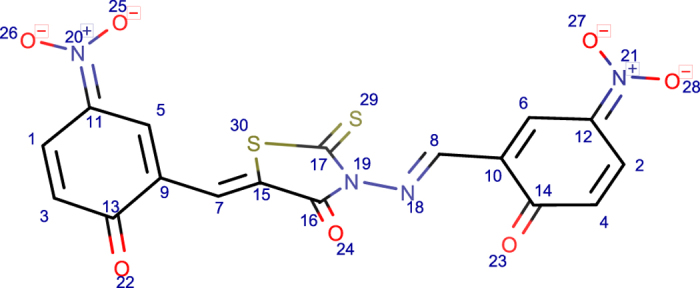
Inconsistency in cis- and trans- configuration. Representation of the 3D structure archived for the PubChem entry CID 1551886, which shows a defined stereochemistry about the double bond between C8 and N18. However, the InChI string archived for this entry “InChI = 1 S/C17H10N4O7S2/c22-13-3-1-11(20(25)26)5-9(13)7-15-16(24)19(17(29)30-15)18-8-10-6-12(21(27)28)2-4-14(10)23/h1-8,22-23 H/p-2**/b15-7-,18-8?**” denotes an ambiguous orientation around the double bond between C8 and N18. As a result, the InChI string generated from the structure by ALATIS failed to match the archived InChI string.

The orientation of the structure of a compound about a double bond, whether the configuration is *cis* or *trans*, is captured precisely in standard InChI strings. These orientations, which can only be identified in 3D structures, are indicated in the “/b” layer of InChI strings. The PubChem compound shown in Figure 5 displays an example of a discrepancy between the configuration of the archived 3D structure and its associated InChI string. In this example, the InChI string of PubChem entry (CID 1551886) contains a question mark in its “/b” layer, which indicates that the configuration of the compound is ambiguous. However, the archived 3D structure represents the *trans* configuration of the compound.

##### (c.2) Inconsistency in stereochemistry of chiral centers

**Figure 6:**
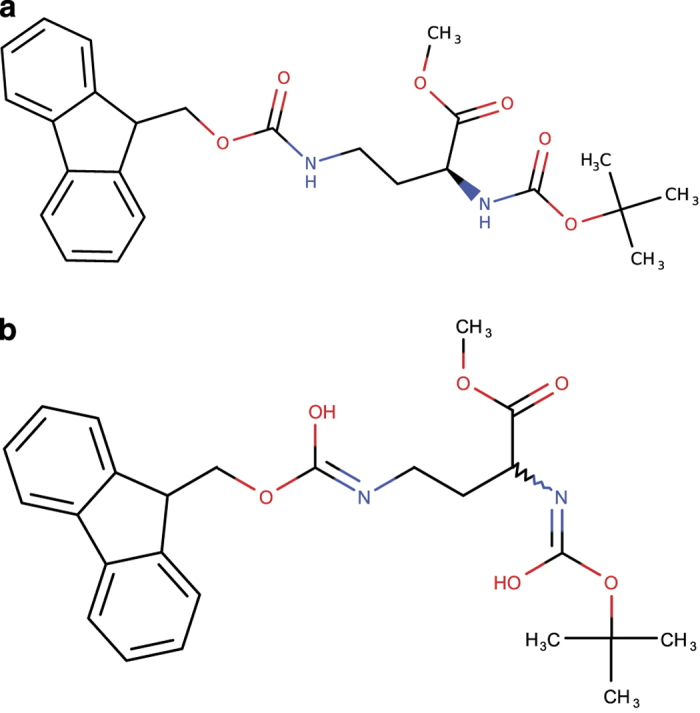
Inconsistency in compound chirality. Example from PubChem CID 130156427. (**a**) Deposited 3D structure of the compound. (**b**) Structure generated from the deposited InChI string. The wavy bond in (**b**) indicates an ambiguous bond chirality. This is because the archived InChI string for this compound “InChI = 1 S/C25H30N2O6/c1-25(2,3)33-24(30)27-21(22(28)31-4)13-14-26-23(29)32-15-20-18-11-7-5-9-16(18)17-10-6-8-12-19(17)20/h5-12,20-21 H,13-15H2,1-4H3,(H,26,29)(H,27,30)” lacks the necessary stereochemistry layer (“/t”). This InChI layer can be found in the standard InChI string reported by ALATIS: “InChI = 1 S/C25H30N2O6/c1-25(2,3)33-24(30)27-21(22(28)31-4)13-14-26-23(29)32-15-20-18-11-7-5-9-16(18)17-10-6-8-12-19(17)20/h5-12,20-21 H,13-15H2,1-4H3,(H,26,29)(H,27,30)/**t21-**/m0/s1.”

The stereochemistry (chirality) of small molecules plays a vital role in determining their function. Among the more than 91 million PubChem entries with 3D structures, our computations using ALATIS indicated that more than 55% of the entries (50,508,180 entries) contained at least one chiral center. About 60% of these entries (30,236,352 entries) were flagged during our analysis, owing to inconsistencies between the stereochemistry layer of the deposited InChI strings in PubChem and those generated by ALATIS from the structures. The complete list of these entries is accessible from the ALATIS website. Figure 6 shows one example from these flagged entries.

## Discussion

The unique representation of chemical compounds and their constituent atoms is of paramount importance to the correct functioning of biochemical databases, including correct storage and retrieval of the compounds and their associated metadata. Unique IDs are essential to the creation and maintenance of cross-references between different databases and in the detection of discrepant data. The ALATIS naming system, which is based on the standard InChI string for a compound generated from its 3D structure, was utilized previously to evaluate the consistency of contents of three reference small molecule databases (BMRB, HMDB, and PDB Ligand Expo)^5^. The ALATIS naming system has subsequently been fully implemented in the Biological Magnetic Resonance data Bank (BMRB) and has been recently adopted by the NMReData initiative^14^. In the work described here, we utilized the powerful hardware capabilities of the NMRbox project to process the PubChem Compound 3D dataset with ALATIS software to further expand the domain of accurate and reproducible compound and atom identifiers of small molecules. The PubChem database provides an important share of information routinely utilized in biomedical investigations of small molecules. Therefore, the accuracy and consistency of the deposited information and the capability of cross-referencing compounds from other databases to PubChem has a direct effect on the cogency of results in biomedical investigations that utilize PubChem.

For entries with stored 3D structures, which covers the vast majority (97.34%) of compounds in PubChem, our analysis created unique compound identifiers (standard InChI) and InChI-based identifiers for all atoms. In addition, we were able to identify PubChem entries with InChI strings that failed to match those generated from the archived structures (about 33% of those analyzed). This information is available for further curation of this key reference database.

## Methods

**Figure 7:**
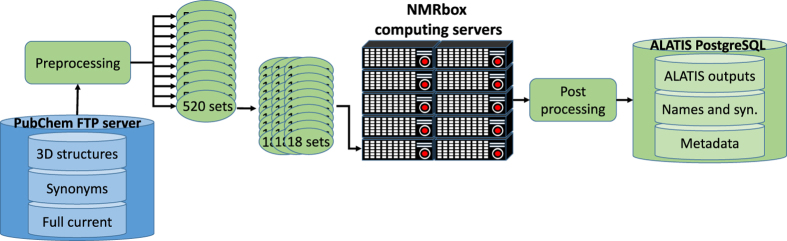
Workflow for utilizing ALATIS for processing PubChem entries. A preprocessing step optimized the computational load by dividing the input data into subsets. Once all entries were processed and unique ALATIS names were established, a post processing step created each entry record for storage in the database.

We defined and programmed a workflow (Fig. 7) and used it to process and analyze the entire set of 3D structure files in PubChem. Because the workflow has been fully defined, it ensures its reproducibility and availability as needed for future reprocessing of the database.

The three-dimensional structure files of the entire PubChem were downloaded in SDF format from the PubChem FTP server on 20 December 2017. At the time of this analysis, the entries consisted of 5,260 compressed SDF files, each containing approximately 25,000 PubChem 3D compound structures. Non-standard cases were handled through processing scripts designed for each specific case. One such script dealt with instances in which the structure file of a compound had been updated on several occasions at different times, and all previous structures for the compound had been preserved in the compressed downloadable file. These obsoleted structures did not have an impact on the results of our analysis, but they increased the computational load because additional structures needed to be processed.

In order to process the large number of entries in a reasonable time, we designated ten servers from the NMRbox platform for the computational task. Each server provided 30 CPU cores (for a total of 300 computing cores). A preprocessing module was developed to split the input SDF files such that 18 SDF files were assigned to one CPU core.

We implemented a distributed computing paradigm wherein ALATIS binary files were executed as a batch job on the correspondingly assigned set of SDF files. The size of the data archive made it impractical to store the reprocessed data (ALATIS output) and associated information as individual files on local storage devices. Therefore, we utilized PostgreSQL open source database [https://www.postgresql.org/] to archive the data. We designed a relational database with three tables: **(i) ALATIS outputs**. This table consists of 11 columns for compound IDs (CID), input structure file, output in SDF format, output in PDB format, output in XYZ format, standard InChI string, formula as generated by ALATIS, warning file, error file, mapping between the input and output atom labels, and an additional map file for the cases that the input structure file contains multiple compounds^5^. **(ii) PubChem metadata**. This table contains four columns for storing the CIDs, molecular formula, molecular weight, and compound’s mass. **(iii) PubChem names.** This table provides the association between the CID and the compound names and synonyms.

The ALATIS website is equipped with a search engine for these tables, and users can query on a PubChem CID or compound name. We note that PubChem does not provide a specific name for a compound: instead, the synonym file contains a list of synonyms for every PubChem CID. Because these lists may contain several hundred synonyms for a compound (for example, PubChem CID 23978 lists 2,286 synonyms), it is not time-efficient to search the entire lists of synonyms when users query a compound name. Therefore, in order to provide a fast search engine, we utilize only the first provided synonym for each entry as the representative name of the compound. However, the complete list of synonyms for each entry is provided on the associated ALATIS webpage.

Because the database contains more than 91 million entries, it was not practical to pre-generate the entry webpages for storage. Instead, we utilized the Jinja2 templating engine [http://jinja.pocoo.org/] to organize and display ALATIS webpages. This template-based web design allows us to create the webpage for an entry from the PostgreSQL database upon request, without the need to physically archive the webpages.

### Code availability

ALATIS is available as a public web-service [http://alatis.nmrfam.wisc.edu/] that runs on a high-throughput computing platform^15^. The executable binary of the software program is available through NMRbox project^11^, and the source codes are available through GitHub [https://github.com/htdashti/ALATIS].

### Data availability

Unique compound identifiers (standard InChI) and all atom identifiers for the analyzed PubChem entries are available through a search engine on the ALATIS website [http://alatis.nmrfam.wisc.edu/]. The processed entries are also available in (Data Citation 1).

## Additional information

**How to cite this article**: Dashti, H. *et al*. Automated evaluation of consistency within the PubChem Compound database. *Sci. Data*. 6:190023 https://doi.org/10.1038/sdata.2019.23 (2019).

**Publisher’s note**: Springer Nature remains neutral with regard to jurisdictional claims in published maps and institutional affiliations.

## Figures and Tables

**Table 1:** Categories and numbers of flagged PubChem entries.

Discrepancy category	InChI layer indicator	Num. flagged PubChem IDs
Atom connectivity	/c	4
	/h	5
Charge	/p	211
	/q	211
Stereochemistry	/b	2,258,069
	/t	30,236,352
